# Systolic Blood Pressure Lower than Heart Rate upon Arrival at and Departure from the Emergency Department Indicates a Poor Outcome for Adult Trauma Patients

**DOI:** 10.3390/ijerph13060528

**Published:** 2016-05-25

**Authors:** Wei-Hung Lai, Shao-Chun Wu, Cheng-Shyuan Rau, Pao-Jen Kuo, Shiun-Yuan Hsu, Yi-Chun Chen, Hsiao-Yun Hsieh, Ching-Hua Hsieh

**Affiliations:** 1 Department of Trauma Surgery, Kaohsiung Chang Gung Memorial Hospital and Chang Gung University College of Medicine, Kaohsiung City 833, Taiwan; abdiel@cgmh.org.tw (W.-H.L.); ah.lucy@hotmail.com (S.-Y.H.); libe320@yahoo.com.tw (Y.-C.C.); sylvia19870714@hotmail.com (H.-Y.H.); 2 Department of Anesthesiology, Kaohsiung Chang Gung Memorial Hospital and Chang Gung University College of Medicine, Kaohsiung City 833, Taiwan; shaochunwu@gmail.com; 3 Department of Neurosurgery, Kaohsiung Chang Gung Memorial Hospital and Chang Gung University College of Medicine, No.123, Ta-Pei Road, Niao-Song District, Kaohsiung City 833, Taiwan; ersh2127@cloud.cgmh.org.tw; 4 Department of Plastic and Reconstructive Surgery, Kaohsiung Chang Gung Memorial Hospital and Chang Gung University College of Medicine, Kaohsiung City 833, Taiwan; bow110470@gmail.com

**Keywords:** reverse shock index (RSI), shock index (SI), injury severity score (ISS), length of stay (LOS), mortality

## Abstract

***Background:*** Hemorrhage is a leading cause of preventable trauma death. In this study, we used the reverse shock index (RSI), a ratio of systolic blood pressure (SBP) to heart rate (HR), to evaluate the hemodynamic stability of trauma patients. As an SBP lower than the HR (RSI < 1) may indicate hemodynamic instability, the objective of this study was to assess the associated complications in trauma patients with an RSI < 1 upon arrival at the emergency department (ED) (indicated as (A)RSI) and at the time of departure from the ED (indicated as (L)RSI) to the operative room or for admission. ***Methods:*** Data obtained from all 16,548 hospitalized patients recorded in the trauma registry system at a Level I trauma center between January 2009 and December 2013 were retrospectively reviewed. A total of 10,234 adult trauma patients aged ≥20 were enrolled and subsequently divided into four groups: Group I, (A)RSI ≥ 1 and (L)RSI ≥ 1 (*n* = 9827); Group II, (A)RSI ≥ 1 and (L)RSI < 1 (*n* = 76); Group III, (A)RSI < 1 and (L)RSI ≥ 1 (*n* = 251); and Group IV, (A)RSI < 1 and (L)RSI < 1 (*n* = 80). Pearson’s χ^2^ test, Fisher’s exact test, or independent Student’s *t*-test was conducted to compare trauma patients in Groups II, III, and IV with those in Group I. ***Results:*** Patients in Groups II, III, and IV had a higher injury severity score and underwent a higher number of procedures, including intubation, chest tube insertion, and blood transfusion, than Group I patients. Additionally, patients of these groups had increased hospital length of stay (16.3 days, 14.9 days, and 22.0 days, respectively), proportion of patients admitted to the intensive care unit (ICU) (48.7%, 43.0%, and 62.5%, respectively), and in-hospital mortality (19.7%, 7.6%, and 27.5%, respectively). Although the trauma patients who had a SBP < 90 mmHg either upon arrival at or departure from the ED also present a more severe injury and poor outcome, those patients who had a SBP ≥ 90 mmHg but an RSI < 1 had a more severe injury and poor outcome than those patients who had a SBP ≥ 90 mmHg and an RSI ≥ 1. ***Conclusions:*** SBP lower than heart rate (RSI < 1) either upon arrival at or departure from the ED may indicate a detrimental sign of poor outcome in adult trauma patients even in the absence of noted hypotension.

## 1. Background

Uncontrolled bleeding is the leading cause of potentially preventable deaths after severe trauma [1,2]. Hemorrhagic shock accounts for approximately 50% of deaths in the first 24 h after injury [3,4]. In addition, 40% of trauma-related deaths are attributed to uncontrolled hemorrhagic shock or its sequelae (such as multiple organ failure) [5]. Measurements of vital signs such as systolic blood pressure (SBP) and heart rate (HR) are unreliable for identifying hypovolemic shock [6,7]. For example, hypotensive individuals can have normal tissue perfusion and those who have had hypertension can have a normal blood pressure during shock [8,9]. To identify hypovolemic shock in patients with trauma, in 1967, Allgower and Burri introduced the concept of shock index (SI), which is the ratio of HR to SBP [10]. The SI is known to be a capable measure for hemodynamic instability [11,12,13] and a clinical indicator of hypovolemic shock upon arrival at the emergency department (ED), with respect to transfusion requirements and hemostatic resuscitation [14]. Published studies to date suggest that an SI ≥ 1 generally indicates an uncompensated shock state that may require further resuscitation [15,16]. Moreover, SI was shown to be a strong predictor of postintubation hemodynamic instability at the ED [17]. Patients with an SI ≥ 1 had a significantly higher transfusion requirement and a higher mortality rate than other major trauma patients, despite prehospital crystalloid resuscitation [11]. In addition, an SI ≥ 1 is associated with an adjusted odds ratio (AOR) of 10.5 (95% confidence interval (CI): 9.3–11.7) for 30-day mortality [18].

An SI ≥ 1 indicates HR ≥ SBP. Although the SI is a useful indicator of shock status of trauma patients, the calculation of the SI as the ratio of HR to SBP contradicts the basic concept of shock, which is generally accompanied by a decreased SBP. In a hemodynamically unstable patient, the SBP is lower than the HR, but not a HR being elevated higher than the SBP, which is revealed as the ratio of the SI. Therefore, we prefer to use the reverse shock index (RSI), the ratio of SBP to HR, to evaluate the hemodynamic stability of trauma patients. An RSI < 1 indicates that the SBP is decreased and lower than the HR and the patient is in a potential state of shock. One of the major benefits of using the RSI for evaluation in the ED is that it can be used quickly without any additional calculation or equipment, especially in crowded EDs where patients may have to wait for hours before evaluation by a physician [19]. An SBP lower than HR (RSI < 1) may alert trauma physicians in a timely manner to recognize the potential hemodynamic instability, allow early intervention, and monitor the response of the resuscitation. Therefore, this study was designed to evaluate the associated outcome of patients with SBP < HR (*i.e.*, RSI < 1) upon arrival at the ED (indicated as (A)RSI) and at the time of departure from the ED (indicated as (L)RSI) to the operative room or for admission, using data from the trauma registry system collected over a five-year period at a Level I trauma center.

## 2. Methods

### 2.1. Ethics Statement

The Chang Gung Memorial Hospital Institutional Review Board (IRB) approved this study (approval number 104-0582B). Informed consent was waived according to the IRB regulations.

### 2.2. Study Design

This retrospective study was conducted at the Kaohsiung Chang Gung Memorial Hospital, a 2400-bed facility and Level I regional trauma center that provides care to trauma patients primarily from southern Taiwan. This study reviewed all 16,548 hospitalized and registered patients added to the Trauma Registry System between 1 January 2009 and 31 December 2013. Because children have a normally high HR, only patients aged ≥20 were included in this study. Patients who had incomplete data were also excluded. The RSI was calculated as the ratio of SBP to HR (RSI = SBP/HR). In total, 10,234 adult trauma patients were enrolled in this study and were then divided into four groups: Group I, (A)RSI ≥ 1 and (L)RSI ≥ 1 (*n* = 9827); Group II, (A)RSI ≥ 1 and (L)RSI < 1 (*n* = 76); Group III, (A)RSI < 1 and (L)RSI ≥ 1 (*n* = 251); and Group IV, (A)RSI < 1 and (L)RSI < 1 (*n* = 80). Group I (stable throughout: patients with a stable hemodynamic status upon both arrival at and departure from the ED) was used as a reference for comparison with Group II (decompensating (stable to unstable): patients with a stable hemodynamic status upon arrival at the ED but whose condition worsened during departure from the ED); Group III (improving (unstable to stable): patients with hemodynamic instability upon arrival at the ED but with an improved condition at the time of departure from the ED); and Group IV (unstable throughout: patients with hemodynamic instability upon both arrival at and during departure from the ED). Additionally, these 10,234 trauma patients were further divided into four additional groups according to their SBP: Group V, (A)SBP ≥ 9 0 mmHg and (L)SBP ≥ 90 mmHg (*n* = 9960); Group VI, (A)SBP ≥ 90 mmHg and (L)SBP < 90 mmHg (*n* = 35); Group VII, (A)SBP < 90 mmHg and (L)SBP ≥ 90 mmHg (*n* = 191); and Group VIII, (A)SBP < 90 mmHg and (L)SBP < 90 mmHg (*n* = 48). Patients leaving the ED were further transferred to the ward, intensive care unit (ICU), or the operative room.

Detailed patient information was collected with regard to age, sex, vital signs (assessed by the physician upon arrival at and departure from the ED), the Glasgow coma scale (GCS) score (assessed upon arrival at the ED), procedures performed by the physician at the ED (intubation, chest tube insertion, and blood transfusion), the abbreviated injury scale (AIS) score for each body region, the injury severity score (ISS), the new injury severity score (NISS), the trauma and injury severity score (TRISS), length of stay (LOS) in the hospital, LOS in the ICU, in-hospital mortality, and complications associated with injuries. In our study, the primary outcome was in-hospital mortality, and the secondary outcomes were the proportion of patients admitted into the ICU, the LOS in the hospital, and the LOS in the ICU.

Data were compared using SPSS version 20 statistical software (IBM Corporation, Armonk, NY, USA). We used a Pearson’s χ^2^ test, Fisher’s exact test, or independent Student’s *t*-test, as applicable. All results are presented as mean ± standard error. A *p*-value of <0.05 was considered statistically significant. A single logistic model was employed to simultaneously estimate the relationship between the independent variables and the outcome. Odds ratios (ORs) were calculated with 95% confidence intervals (CIs). To estimate associations between different groups and mortality outcomes, we created separate logistic regression models controlled by the most important confounder—ISS—and expressed AORs for mortality with 95% CIs.

## 3. Results

### 3.1. Injury Characteristics of Patients Divided According to Reverse Shock Index (RSI)

The mean ages of patients in Groups I, II, III, and IV were 42.9 ± 13.4, 39.3 ± 13.7, 39.4 ± 12.6, and 38.2 ± 12.7 years, respectively (Table 1). Compared to Group I patients, Group II, III, and IV patients had a significantly younger population. A statistically significant male predominance was found in both Group III and IV patients as compared to Group I patients. There were also significantly lower GCS scores of more than 1 point in Group II, III, and IV patients than in Group I patients. In addition, the distribution of scores (GCS ≤ 8, 9–12 or ≥ 13) were different between patients of Groups II, III, and IV and those of Group I. Analysis of AIS scores revealed that, compared to Group I patients, Group II patients had sustained significantly higher rates of injuries to the head/neck, face, thorax, and abdomen, but not to the extremities; Group III patients had sustained significantly higher rates of injuries to the head/neck, face, thorax, abdomen and extremities; and Group IV patients had sustained significantly higher rates of injuries to the head/neck, thorax, abdomen and extremities. A significantly higher ISS score was found in Group II, III, and IV patients in comparison to Group I patients. When stratified by injury severity (ISS of <16, 16–24, or ≥25), Groups II, III, and IV had more patients with an ISS of 16–24 and ≥25 and had less patients with an ISS of <16 than Group I patients. In addition, we also found a significantly higher NISS in Group II, III, and IV patients than in Group I patients. Further, Group I patients showed a significantly lower TRISS than Group II, III, and IV patients. The in-hospital mortality rate of Group II (19.7%; *p* < 0.001), III (7.6%; *p* < 0.001), and IV (27.5%; *p* < 0.001) patients were significantly higher than that of the Group I patients (0.9%). After adjustment by ISS, the AOR of mortality of the Group II, III, and IV patients was 7.6-fold (95% confidence interval (CI): 3.5–16.4; *p* < 0.001), 2.0-fold (95% CI: 1.0–3.8; *p* = 0.047), and 9.5-fold (95% CI: 4.9–18.8; *p* < 0.001) greater than that of the Group I patients, respectively.

### 3.2. Injury Characteristics of Patients Divided According to Systolic Blood Pressure (SBP)

Compared to Group V patients, no significant difference in the age and sex was found in Group VI, VII, and VIII patients (Table 2). There were significantly lower GCS scores of more than 1 point in Group VI, VII, and VIII patients as compared to Group V patients. In addition, the distribution of scores (GCS ≤ 8, 9–12 or ≥ 13) were different between patients of Groups VI, VII, and VIII and those of Group V. Analysis of AIS scores revealed that, compared to Group V patients, Group VI patients had sustained significantly higher rates of injuries to the head/neck and thorax, but not to the face, abdomen, and extremities; Group VII patients had sustained significantly higher rates of injuries to the head/neck, thorax, abdomen, and extremities, but not to the face; and Group VIII patients had sustained significantly higher rates of injuries to the thorax and abdomen, but not to the head/neck, face, and extremities. A significantly higher ISS score was found in Group VI, VII, and VIII patients in comparison to Group V patients. When stratified by injury severity (ISS of <16, 16–24, or ≥25), the Group VI, VII, and VIII patients had more patients with an ISS of 16–24 and ≥25 and had less patients with an ISS of <16 than Group V patients. In addition, we found a significantly higher NISS in Group VI, VII, and VIII patients than in Group V patients. Furthermore, Group V patients showed a significantly lower TRISS than Group VI, VII, and VIII patients. The in-hospital mortality rate of Group VI (31.4%; *p* < 0.001), VII (8.9%; *p* < 0.001), and VIII (25.0%; *p* < 0.001) patients were significantly higher than that of the Group V patients (1.1%). After adjustment by ISS, the AOR of mortality of the Group VI, VII, and VIII patients was 28.1-fold (95% confidence interval (CI): 11.6–68.2; *p* < 0.001), 3.7-fold (95% CI: 1.9–7.6; *p* < 0.001), and 11.1-fold (95% CI: 4.9–25.5; *p* < 0.001) greater than that of the Group V patients, respectively.

### 3.3. Management Characteristics of Patients Divided According to the RSI

As shown in Table 3, all Group II, III, and IV patients had a significantly higher odds ratio (OR) for receiving intubation, chest tube insertion, and blood transfusion than Group I patients. A significantly longer hospital LOS was noted among Group II, III, and IV patients compared with Group I patients (16.3, 14.9, and 22.0 *vs.* 8.9 days, respectively; *p* < 0.001). A total of 5203 (52.9%) patients from Group I, 32 (42.1%) patients from Group II, 110 (43.8%) patients from Group III, and 9 (11.3%) patients from Group IV went to the operating room directly from the ED. Furthermore, a significantly larger proportion of Group II, III, and IV patients were admitted to the ICU as compared to Group I patients (48.7%, 43.0%, and 62.5% *vs.* 15.1%, respectively; all *p* < 0.001). A significantly longer ICU LOS was noted in Group IV patients (14.7 *vs.* 8.8 days; *p* < 0.001), but not in Group II and IV patients.

### 3.4. Management Characteristics of Patients Divided According to the SBP

Compared with Group V patients, Group VI patients had a statistically significantly higher OR for receiving intubation and blood transfusion (OR: 4.8 and 8.4, *p* = 0.030 and < 0.001, respectively); Group VII patients had a statistically significantly higher OR for receiving intubation, chest tube insertion, and blood transfusion (OR: 4.7, 5.6, and 16.0, respectively, all *p* < 0.001); and Group VIII patients had a statistically significantly higher OR for receiving intubation, chest tube insertion, and blood transfusion (OR: 11.8, 6.4, and 24.3, *p* < 0.001, *p* = 0.005, and *p* < 0.001, respectively) (Table 4). A significantly longer hospital LOS was noted among Group VII and VIII patients, but not Group VI patients, compared with Group V patients (Group VII and VIII: 12.2 and 21.4 *vs.* Group V: 9.1 days, *p* < 0.001 and *p* = 0.001, respectively). Furthermore, a significantly larger proportion of Group VI, VII, and VIII patients were admitted to the ICU as compared to Group V patients (40.0%, 39.8% and 54.2% *vs.* 15.7%, respectively; all *p* < 0.001), and Group VI, VII, and VIII patients did not show significantly longer ICU LOS than Group V patients.

### 3.5. Injury Characteristics of Patients with SBP ≥ 90 mmHg but RSI < 1

Discrepancy of injury characteristics existed in the subgroups of patients divided by either the RSI or SBP level (Table 5). Upon arrival at the ED, 186 (1.8%) patients had an SBP ≥ 90 mmHg but an RSI < 1, indicating the occurrence of an occult hypoperfusion even in the absence of noted hypotension. However, at the time of departure from the ED, 112 (1.1%) patients had an SBP ≥ 90 mmHg but an RSI < 1. As shown in Table 6, further analysis compared the patients with SBP ≥ 90 mmHg but RSI < 1 to those who had a recognized stable hemodynamic condition (*i.e.*, RSI ≥ 1 and SBP ≥ 90 mmHg) and revealed that regardless of arrival at the ED or departure from the ED, a significantly lower GCS score, higher proportion of patients in comatose status (GCS ≤ 8), higher rates of injuries to the head/neck, face, thorax, and abdomen, but lower rate of injures to the extremities, higher ISS score, more patients with an ISS of 16–24 and ≥25 and less patients with an ISS of < 16, and higher in-hospital mortality rate were found. After adjustment with ISS, the AOR of mortality of the group of patients (SBP ≥ 90 mmHg but RSI < 1) was 2.2-fold (95% CI: 1.1–4.5; *p* = 0.036) and 6.2-fold (95% CI: 3.2–11.9; *p* < 0.001) upon arrival at and the time of departure from the ED, respectively, greater than that of the hemodynamic patients. Notably, the number of the patients with poor outcome identified by an RSI < 1 either upon arrival or departure from the ED (total of 407 patients in Levels II + III + IV) was larger than that of patients with poor outcome, identified by an SBP < 90 mmHg (total of 274 patients in Levels VI + VII + VIII), indicating that RSI may be more sensitive than SBP for identifying the patients at risk of poor outcome in the ED.

### 3.6. Management Characteristics of Patients with SBP ≥ 90 mmHg but RSI < 1

Compared to the hemodynamically stable patients who had an RSI ≥ 1 and an SBP ≥ 90 mmHg, those who had an SBP ≥ 90 mmHg but an RSI < 1 (Table 7) presented a significantly higher OR for receiving intubation, chest tube insertion, and blood transfusion (OR: 5.5, 7.3, and 8.2, respectively, all *p* < 0.001), longer hospital LOS (16.6 *vs.* 9.0 days; *p* < 0.001), proportion of patients admitted to the ICU (43.5% *vs.* 15.3%; *p* < 0.001), and ICU LOS (13.2 *vs.* 8.7 days; *p* < 0.001).

### 3.7. Characteristics of Associated Injuries of Patients Divided According to the RSI

**Figure 1:**
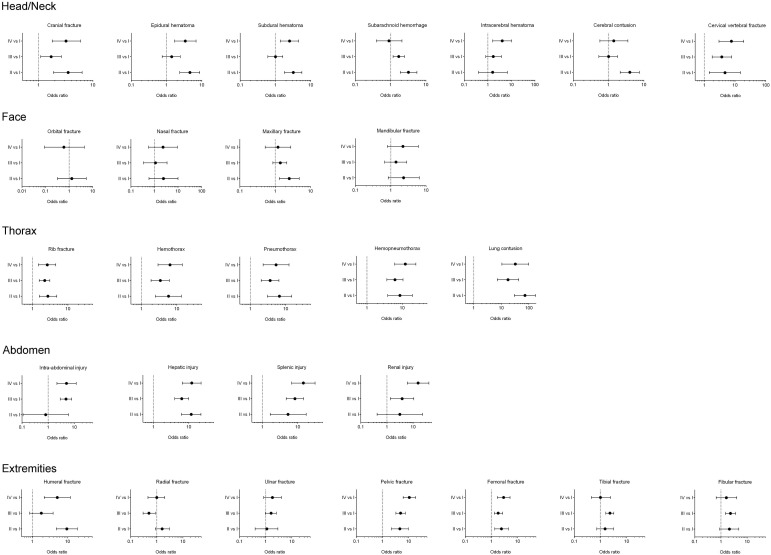
The odds ratio of the associated injuries of hospitalized trauma patients in Groups II, III, and IV *vs.* trauma patients in Group I.

In the head/neck region, all three groups (II, III, and IV) had a significantly higher OR for sustaining cranial fracture and cervical vertebral fracture than Group I patients. In the thoracic region, all three groups had a significantly higher OR for sustaining rib fracture, hemothorax, pneumothorax, hemopneumothorax, and lung contusion. In the abdomen, all three groups had a significantly higher OR for sustaining hepatic injury and splenic injury. In the extremity region, all three groups had a significantly higher OR for sustaining pelvic fracture and femoral fracture. In addition, all three groups showed significantly higher ORs for sustaining several associated injuries as compared to Group I patients; detailed information on this is listed in Figure 1 and Table A1.

## 4. Discussion

SI is a known predictor of mortality and adverse outcomes in trauma patients [20]. In prior reports, patients with an SI ≥ 1 had a higher mortality rate than other major trauma patients [10,11,21] and such an SI presented as the strongest predictor for mortality (OR: 3.1; 95% CI: 2.6–3.3; *p* = 0.001) in a review of 485,595 geriatric trauma patients [22]. In addition, an SI ≥ 1 is associated with a higher 30-day mortality (AOR: 10.5; 95% CI: 9.3–11.7) [18]. In this study, an SBP lower than HR (*i.e.*, RSI < 1) yields an SI ≥ 1. Therefore, as expected, in trauma patients, an RSI < 1 either upon arrival at or departure from the ED indicated a more severe injury and poor outcome. Group II, III, and IV patients had a higher ISS and underwent more procedures than Group I patients; they also had increased hospital LOS, increased admission to the ICU, and in-hospital mortality. Moreover, they had a higher incidence of commonly associated injuries, particularly severe injury to the thoracoabdominal areas that could cause significant blood loss, including rib fracture, hemothorax, pneumothorax, hemopneumothorax, lung contusion, hepatic injury, and splenic injury. In addition, the trauma patients who had an SBP < 90 mmHg either upon arrival at or departure from the ED presented more severe injury and poor outcome than those patients who had an SBP ≥ 90 mmHg. Group VI, VII, and VIII patients had a higher ISS and underwent more procedures compared to Group V patients; they also had the worst outcomes of the patients admitted to the ICU and in-hospital mortality. However, those patients who had an SBP ≥ 90 mmHg but an RSI < 1 either upon arrival at or departure from the ED presented more severe injury and poor outcome than those hemodynamically stable patients, revealing that even though no hypotension was noted, an RSI < 1 may indicate an occult sign of the deteriorated physiological condition. In addition, the number of patients with poor outcome identified by an RSI < 1 either upon arrival at or departure from the ED was larger than those patients with poor outcome identified by an SBP < 90 mmHg, indicating that RSI may be more sensitive than SBP for identifying patients at risk of poor outcome in the ED.

In Group II patients who had a stable hemodynamic status upon arrival at the ED but whose condition worsened at departure from the ED, a 7.6-fold higher odds of mortality was found as compared to Group I patients. The deterioration of a stable hemodynamic status during the stay at the ED may imply an inadequate resuscitation, a collapse of the physiological reserve after the trauma, or unidentified associated hemorrhage. In addition to the abovementioned injures to the thoracoabdominal areas as well as pelvic and femoral fractures, a higher OR for sustaining cranial fracture (3.5-fold), epidural hematoma (4.6-fold), subdural hematoma (3.2-fold), subarachnoid hemorrhage (3.2-fold), cerebral contusion (4.0-fold), cervical vertebral fracture (4.7-fold), maxillary fracture (2.5-fold), and humeral fracture (9.5-fold) was noted among Group II patients as compared to Group I patients. In addition, nearly half the patients (48.7%) in Group II required admission to the ICU. The higher mortality rate found in Group II patients indicates that patients who have a stable hemodynamic condition upon arrival at the ED but experience a rapid deterioration of hemodynamic status at the ED require specific attention. Young patients who present with tachycardia and mild hypotension are in danger of compensatory mechanism failure and may slip into profound shock unless vigorous therapy is initiated [23]. In these patients, reliance on SBP alone may delay recognition of the shock state [23]. In addition, in Group III patients who had hemodynamic instability upon arrival at the ED but whose condition improved at the time of departure, an improved condition was noted after resuscitation during the stay at the ED, though there was a 2.0-fold higher odds of mortality as compared to Group I patients, and 43% of Group III patients required admission to the ICU.

Among all these patients, Group IV patients who had hemodynamic instability at both arrival at and departure from the ED are of prime focus, because they had worse outcomes compared to patients in the other groups. The Group IV patients, albeit younger, had a 9.5-times greater AOR for mortality than Group I patients. These patients also had a longer LOS in the hospital (22.0 days) and ICU (14.7 days) as well as a higher proportion of patients admitted into ICU (62.5%). In addition to the injures to the thoracoabdominal areas as well as pelvic and femoral fracture, a higher OR for sustaining cranial fracture (3.2-fold), epidural hematoma (3.4-fold), subdural hematoma (2.5-fold), intracerebral hematoma (4.1-fold), cervical vertebral fracture (7.6-fold), and humeral fracture (5.1-fold) was noted among Group II patients than among Group I patients. Currently, although it is essential to stabilize the patient’s hemodynamic status before transfer to avoid circulatory complications, strong evidence defining the optimal blood pressure level during active hemorrhagic shock or resuscitation is lacking in the literature [24,25]. High-volume fluid resuscitation with crystalloids has been replaced with damage control resuscitation (DCR), which consists of hypothermia prevention, permissive hypotension (SBP targeted at 90 mmHg in bleeding patients without head injury), immediate administration of blood products, prevention of crystalloid and colloid use, and damage control surgery or angioembolization to treat the cause of bleeding [26,27,28,29]. For patients with trauma but without brain injury, the European guidelines recommend a target SBP of 80–90 mmHg until major bleeding in the initial phase has been stopped [30]. The DCR strategies have generally been performed at the ED for more than a decade in our hospital and the additional use of an RSI < 1 to monitor the hemodynamic status of the patients may have the potential to identify some subjects with occult hypotension among these trauma patients.

Despite the important findings, our study has a few limitations. First, as mentioned above, the data were collected prospectively as a part of the required trauma registry process, but our analyses were performed retrospectively and are thus subject to the limitations of all retrospective studies. Because of the retrospective nature of the study, we were unable to clarify the cause−effect relationship of massive bleeding and mortality; however, even a hypoperfusion status may not cause mortality directly, although it may have some detrimental effects on the distal important vital organs. In addition, the lack of available data regarding the factors influencing the decision-making regarding patient management such as the requirement of operation, blood transfusion, or admission into the ICU may have biased the outcome. Second, injured patients who did not survive at hospital arrival or who were discharged from the ED were not included in the sample, which could result in a selection bias. Third, because the patients’ HR or SBP fluctuated with each minute, the values of RSI changed accordingly. Some borderline patients may develop an RSI < 1 or > 1 at some time points in the ED, making the assignment of some patients to a group debatable. Fourth, the impact of pre-existing comorbidities and the lack of available data regarding patient management in the course of hospitalization was not included and may have resulted in a bias in the outcome. Finally, some important data, such as those regarding cost, complications, and rehabilitation process, were not assessed and may have limited the outcome evaluation results.

## 5. Conclusions

This retrospective analysis spanning a five-year period showed that using a reverse shock index RSI < 1 as a threshold to evaluate the hemodynamic stability of trauma patients during the emergency department (ED) stage can identify poor outcomes in certain subgroups of patients in the ED setting. As a reverse value of the shock index (SI) and being mathematically equivalent, RSI is expected to express a similar usefulness as the SI; however, this study replaced SI with RSI intentionally to illustrate that the trauma patients whose systolic blood pressure (SBP) was lower than heart rate (HR) upon arrival at and departure from the ED, *i.e.*, RSI < 1, had a poor outcome and required special attention.

## Figures and Tables

**Table 1:** Demographic and injury characteristics of hospitalized trauma patients divided by the level of RSI.

Variables	(A)RSI ≥ 1 (L)RSI < 1 *n* = 76 (II)	(A)RSI < 1 (L)RSI ≥ 1 *n* = 251 (III)	(A)RSI < 1 (L)RSI < 1 *n* = 80 (IV)	(A)RSI ≥ 1 (L)RSI ≥ 1 *n* = 9827 (I)	II *vs.* I		III *vs.* I		IV *vs.* I	
					OR (95% CI)	*p*	OR (95% CI)	*p*	OR (95% CI)	*p*
Age	39.3 ± 13.7	39.4 ± 12.6	38.2 ± 12.7	42.9 ± 13.4	-	0.020	-	<0.001	-	0.002
Gender										
Male	48 (63.2)	177 (70.5)	63 (78.8)	6193 (63.0)	1.0 (0.6–1.6)	0.980	1.4 (1.1–1.9)	0.015	2.2 (1.3–3.7)	0.004
Female	28 (36.8)	74 (29.5)	17 (21.2)	3634 (37.0)	1.0 (0.6–1.6)	0.980	0.7 (0.5–0.9)	0.015	0.5 (0.3–0.8)	0.004
GCS, mean ± SD	12.0 ± 4.5	12.5 ± 4.2	11.2 ± 4.7	14.4 ± 2.0	-	<0.001	-	<0.001	-	<0.001
GCS, median (IQR)	15 (8–15)	15 (12–15)	14 (6–15)	15 (15–15)						
≤8	21 (27.6)	50 (19.9)	22 (27.5)	373 (3.8)	9.7 (5.8–16.2)	<0.001	6.3 (4.6–8.7)	<0.001	9.6 (5.8–15.9)	<0.001
9–12	4 (5.3)	20 (8.0)	12 (15.0)	319 (3.2)	1.7 (0.6–4.6)	0.324	2.6 (1.6–4.1)	<0.001	5.3 (2.8–9.8)	<0.001
≥13	51 (67.1)	181 (72.1)	46 (57.5)	9135 (93.0)	0.2 (0.1–0.3)	<0.001	0.2 (0.2–0.3)	<0.001	0.1 (0.1–0.2)	<0.001
AIS										
Head/Neck	37 (48.7)	99 (39.4)	35 (43.8)	2331 (23.7)	3.1 (1.9–4.8)	<0.001	2.1 (1.6–2.7)	<0.001	2.5 (1.6–3.9)	<0.001
Face	26 (34.2)	59 (23.5)	18 (22.5)	1777 (18.1)	2.4 (1.5–3.8)	<0.001	1.4 (1.0–1.9)	0.028	1.3 (0.8–2.2)	0.307
Thorax	30 (39.5)	76 (30.3)	31 (38.8)	1133 (11.5)	5.0 (3.2–8.0)	<0.001	3.3 (2.5–4.4)	<0.001	4.9 (3.1–7.6)	<0.001
Abdomen	17 (22.4)	64 (25.5)	26 (32.5)	587 (6.0)	4.5 (2.6–7.8)	<0.001	5.4 (4.0–7.2)	<0.001	7.6 (4.7–12.2)	<0.001
Extremities	49 (64.5)	162 (64.5)	50 (62.5)	7139 (72.6)	0.7 (0.4–1.1)	0.112	0.7 (0.5–0.9)	0.005	0.6 (0.4–1.0)	0.043
ISS, mean ± SD	17.8 ± 13.2	14.7 ± 13.8	21.4 ± 14.4	7.7 ± 6.6	-	<0.001	-	<0.001	-	<0.001
ISS, median (IQR)	17 (9–25)	10 (4–18)	20 (10–29)	5 (4–9)						
<16	37 (48.7)	154 (61.4)	30 (37.5)	8611 (87.6)	0.1 (0.1–0.2)	<0.001	0.2 (0.2–0.3)	<0.001	0.1 (0.1–0.1)	<0.001
16–24	20 (26.3)	54 (21.5)	20 (25.0)	878 (8.9)	3.6 (2.2–6.1)	<0.001	2.8 (2.1–3.8)	<0.001	3.4 (2.0–5.7)	<0.001
≥25	19 (25.0)	43 (17.1)	30 (37.5)	338 (3.4)	9.4 (5.5–15.9)	<0.001	5.8 (4.1–8.2)	<0.001	16.8 (10.6–26.8)	<0.001
NISS	19.4 ± 13.5	16.1 ± 14.6	25.6 ± 17.1	9.0 ± 8.3	-	<0.001	-	<0.001	-	<0.001
Mortality	15 (19.7)	19 (7.6)	22 (27.5)	89 (0.9)	26.9 (14.7–49.1)	<0.001	9.0 (5.4–15.0)	<0.001	41.5 (24.4–70.7)	<0.001
Controlled by ISS	-	-	-	-	7.6 (3.5–16.4)	<0.001	2.0 (1.0–3.8)	0.047	9.5 (4.9–18.8)	<0.001

**Table 2:** Management characteristics of hospitalized trauma patients divided by the level of RSI.

Variables	(A)SBP ≥ 90 (L)SBP < 90 *n* = 35 (VI)	(A)SBP < 90 (L)SBP ≥ 90 *n* = 191 (VII)	(A)SBP < 90 (L)SBP < 90 *n* = 48 (VIII)	(A)SBP ≥ 90 (L)SBP ≥ 90 *n* = 9960 (V)	VI *vs.* V		VII *vs.* V		VIII *vs.* V	
					OR (95% CI)	*p*	OR (95% CI)	*p*	OR (95% CI)	*p*
Age	45.3 ± 15.4	42.1 ± 12.7	43.3 ± 12.4	42.8 ± 13.4	-	0.263	-	0.451	-	0.807
Gender										
Male	25 (71.4)	124 (64.9)	26 (54.2)	6306 (63.3)	1.4 (0.7–3.0)	0.382	1.1 (0.8–1.5)	0.703	0.7 (0.4–1.2)	0.229
Female	10 (28.6)	67 (35.1)	22 (45.8)	3654 (36.7)	0.7 (0.3–1.4)	0.382	0.9 (0.7–1.3)	0.703	1.5 (0.8–2.6)	0.229
GCS, mean ± SD	12.5 ± 4.3	12.7 ± 4.1	11.6 ± 4.5	14.4 ± 2.1	-	0.012	-	<0.001	-	<0.001
GCS, median (IQR)	15 (11–15)	15 (12–15)	14.5 (9–15)	15 (15–15)						
≤8	8 (22.9)	34 (17.8)	10 (20.8)	414 (4.2)	6.8 (3.1–15.1)	<0.001	5.0 (3.4–7.3)	<0.001	6.1 (3.0–12.3)	<0.001
9–12	2 (5.7)	14 (7.3)	9 (18.8)	330 (3.3)	1.8 (0.4–7.4)	0.631	2.3 (1.3–4.0)	0.007	6.7 (3.2–14.0)	<0.001
≥13	25 (71.4)	143 (74.9)	29 (60.4)	9216 (92.5)	0.2 (0.1–0.4)	<0.001	0.2 (0.2–0.3)	<0.001	0.1 (0.1–0.2)	<0.001
AIS										
Head/Neck	17 (48.6)	67 (35.1)	16 (33.3)	2402 (24.1)	3.0 (1.5–5. 8)	0.002	1.7 (1.3–2.3)	0.001	1.6 (0.9–2.9)	0.174
Face	10 (28.6)	36 (18.8)	5 (10.4)	1829 (18.4)	1.8 (0.9–3.7)	0.126	1.0 (0.7–1.5)	0.858	0.5 (0.2–1.3)	0.191
Thorax	15 (42.9)	58 (30.4)	14 (29.2)	1183 (11.9)	5.6 (2.8–10.9)	<0.001	3.2 (2.4–4.4	<0.001	3.1 (1.6–5.7)	0.001
Abdomen	4 (11.4)	61 (31.9)	19 (39.6)	610 (6.1)	2.0 (0.7–5.6)	0.165	7.2 (5.3–9.9)	<0.001	10.0 (5.6–18.0)	<0.001
Extremities	21 (60.0)	125 (65.4)	37 (77.1)	7217 (72.5)	0.6 (0.3–1.1)	0.127	0.7 (0.5–1.0)	0.032	1.3 (0.7–2.5)	0.516
ISS, mean ± SD	15.3 ± 10.1	13.7 ± 13.1	17.9 ± 11.9	7.9 ± 7.0	-	<0.001	-	<0.001	-	<0.001
ISS, median (IQR)	13 (9–22)	9 (4–18)	16.5 (9–26)	5 (4–9)						
<16	18 (51.4)	131 (68.6)	22 (45.8)	8661 (87.0)	0.2 (0.1–0.3)	<0.001	0.3 (0.2–0.5)	<0.001	0.1 (0.1–0.2)	<0.001
16–24	9 (25.7)	33 (17.3)	11 (22.9)	919 (9.2)	3.4 (1.6–7.29)	0.004	2.1 (1.4–3.0)	<0.001	2.9 (1.5–5.8)	0.004
≥25	8 (22.9)	27 (14.1)	15 (31.2)	380 (3.8)	7.5 (3.37–16.6)	<0.001	4.2 (2.7–6.3)	<0.001	11.5 (6.2–21.3)	<0.001
NISS	17.2 ± 10.3	15.5 ± 14.2	21.9 ± 14.6	9.2 ± 8.6	-	<0.001	-	<0.001	-	<0.001
Mortality	11 (31.4)	17 (8.9)	12 (25.0)	105 (1.1)	43.0 (20.5–90.1)	<0.001	9.2 (5.4–15.6)	<0.001	31.3 (15.8–61.8)	<0.001
Controlled by ISS	-	-	-	-	28.1 (11.6–68.2)	<0.001	3.7 (1.9–7.6)	<0.001	11.1 (4.9–25.5)	<0.001

**Table 3:** Demographic and injury characteristics of hospitalized trauma patients divided by the level of SBP.

Variables	(A)RSI ≥ 1 (L)RSI < 1 n = 76 (II)	(A)RSI < 1 (L)RSI ≥ 1 *n* = 251 (III)	(A)RSI < 1 (L)RSI < 1 *n* = 80 (IV)	(A)RSI ≥ 1 (L)RSI ≥ 1 *n* = 9827 (I)	II *vs*. I		III *vs*. I		IV *vs*. I	
					OR (95% CI)	*p*	OR (95% CI)	*p*	OR (95% CI)	*p*
Procedures at ED										
Intubation	13 (17.1)	24 (9.6)	14 (17.5)	168 (1.7)	11.9 (6.4–22.0)	<0.001	6.1 (3.9–9.5)	<0.001	12.2 (6.7–22.2)	<0.001
Chest tube insertion	7 (9.2)	21 (8.4)	10 (12.5)	121 (1.2)	8.1 (3.7–18.1)	<0.001	7.3 (4.5–11.9)	<0.001	11.5 (5.8–22.8)	<0.001
Blood transfusion	14 (18.4)	45 (17.9)	32 (40.0)	184 (1.9)	11.8 (6.5–21.5)	<0.001	11.4 (8.0–16.3)	<0.001	34.9 (21.8–55.9)	<0.001
Hospital LOS (days)	16.3 ± 16.1	14.9 ± 16.7	22.0 ± 20.7	8.9 ± 9.7	-	<0.001	-	<0.001	-	<0.001
ICU LOS										
n (%)	37 (48.7)	108 (43.0)	50 (62.5)	1484 (15.1)	5.3 (3.4–8.4)	<0.001	4.2 (3.3–5.5)	<0.001	9.4 (5.9–14.8)	<0.001
days	6.9 ± 6.2	10.8 ± 20.2	14.7 ± 18.2	8.8 ± 10.5	-	0.282	-	0.070	-	<0.001

**Table 4:** Management characteristics of hospitalized trauma patients divided by the level of SBP.

Variables	(A)SBP ≥ 90 (L)SBP < 90 *n* = 35 (VI)	(A)SBP < 90 (L)SBP ≥ 90 *n* = 191 (VII)	(A)SBP < 90 (L)SBP < 90 *n* = 48 (VIII)	(A)SBP ≥ 90 (L)SBP ≥ 90 *n* = 9960 (V)	VI *vs*. V		VII *vs.* V		VIII *vs.* V	
					OR (95% CI)	*p*	OR (95% CI)	*p*	OR (95% CI)	*p*
Procedures at ED										
Intubation	3 (8.6)	16 (8.4)	9 (18.8)	191 (1.9)	4.8 (1.5–15.8)	0.030	4.7 (2.8–8.0)	<0.001	11.8 (5.6–24.7)	<0.001
Chest tube insertion	2 (5.7)	14 (7.3)	4 (8.3)	139 (1.4)	4.3 (1.02–18.0)	0.087	5.6 (3.2–9.9)	<0.001	6.4 (2.3–18.1)	0.005
Blood transfusion	6 (17.1)	54 (28.3)	18 (37.5)	240 (2.4)	8.4 (3.5–20.4)	<0.001	16.0 (11.4–22.4)	<0.001	24.3 (13.4–44.2)	<0.001
Hospital LOS (days)	11.1 ± 12.5	12.2 ± 11.2	21.4 ± 22.8	9.1 ± 10.1	-	0.347	-	<0.001	-	0.001
ICU LOS										
*n* (%)	14 (40.0)	76 (39.8)	26 (54.2)	1563 (15.7)	3.6 (1.8–7.1)	<0.001	3.6 (2.6–4.8)	<0.001	6.3 (3.6–11.2)	<0.001
days	6.1 ± 7.4	9.4 ± 10.7	14.7 ± 15.7	8.9 ± 11.59	-	0.369	-	0.742	-	0.072

**Table 5:** Group of patients divided by the RSI and SBP level.

Status	Number	RSI < 1, SBP ≥ 90	RSI < 1, SBP < 90	RSI ≥ 1, SBP ≥ 90	RSI ≥ 1, SBP < 90
At ED	n, (%)	186 (1.8%)	145 (1.4%)	9809 (95.9%)	94 (0.9%)
Leave ED	n, (%)	112 (1.1%)	44 (0.4%)	10,039 (98.1%)	39 (0.4%)

**Table 6:** Demographic and injury characteristics of hospitalized trauma patients who had stable SBP but an RSI < 1.

Variables	At ED							Leave ED						
	RSI < 1, SBP ≥ 90 *n* = 186		RSI ≥ 1, SBP ≥ 90 *n* = 9809		OR (95% CI)		*p*	RSI < 1, SBP ≥ 90 *n* = 112		RSI ≥ 1, SBP ≥ 90 *n* = 10,039		OR (95% CI)		*p*
Age	36.6 ± 12.0		42.9 ± 13.4		-		<0.001	36.8 ± 12.7		42.8 ± 13.4		-		<0.001
Gender														
Male	142 (76.3)		6189 (63.1)		1.8 (1.3–2.7)		<0.001	81 (72.3)		6349 (63.2)		1.5 (1.0–2.3)		0.049
Female	44 (23.7)		3620 (36.9)		0.5 (0.4–0.8)		<0.001	31 (27.7)		3690 (36.8)		0.7 (0.4–1.0)		0.049
GCS, mean ± SD	12.6 ± 4.0		14.42.0		-		<0.001	12.0 ± 4.4		14.4 ± 2.1		-		<0.001
GCS, median (IQR)	15 (12–15)		15 (15–15)					15 (8–15)		16 (9–25)				
≤8	35 (18.8)		387 (3.9)		5.6 (3.9–8.3)		<0.001	29 (25.9)		419 (4.2)		8.0 (5.2–12.4)		<0.001
9–12	17 (9.1)		315 (3.2)		3.0 (1.8–5.1)		<0.001	8 (7.1)		336 (3.3)		2.2 (1.1–4.6)		0.036
≥13	134 (72.1)		9107 (92.8)		0.2 (0.1–0.3)		<0.001	75 (67.0)		9284 (92.5)		0.2 (0.1–0.3)		<0.001
AIS														
Head/Neck	78 (41.9)		2341 (23.9)		2.3 (1.7–3.1)		<0.001	51 (45.5)		2418 (24.1)		2.6 (1.8–3.8)		<0.001
Face	52 (28.0)		1787 (18.2)		1.7 (1.3–2.4)		0.001	36 (32.1)		1829 (18.2)		2.1 (1.4–3.2)		<0.001
Thorax	56 (30.1)		1142 (11.6)		3.3 (2.4–4.5)		<0.001	43 (38.4)		1198 (11.9)		4.6 (3.1–6.8)		<0.001
Abdomen	31 (16.7)		583 (5.9)		3.2 (2.1–4.7)		<0.001	25 (22.3)		646 (6.4)		4.2 (2.7–6.6)		<0.001
Extremities	120 (64.5)		7118 (72.6)		0.7 (0.5–0.9)		0.020	69 (61.6)		7273 (72.4)		0.6 (0.4–0.9)		0.011
ISS, mean ± SD	15.6 ± 14.2		7.8 ± 6.7		-		<0.001	18.7 ± 15.0		7.9 ± 7.0		-		<0.001
ISS, median (IQR)	13 (4–22)		5 (4–9)					15 (15–15)		5 (4–9)				
<16	105 (56.5)		8574 (87.4)		0.2 (0.1–0.3)		<0.001	55 (49.1)		8737 (87.0)		0.1 (0.1–0.2)		<0.001
16–24	42 (22.6)		886 (9.0)		2.9 (2.1–4.2)		<0.001	26 (23.2)		926 (9.2)		3.0 (1.9–4.6)		<0.001
≥25	39 (21.0)		349 (3.6)		7.2 (5.0–10.4)		<0.001	31 (27.7)		376 (3.7)		9.8 (6.4–15.1)		<0.001
Mortality	16 (8.6)		100 (1.0)		9.1 (5.3–15.8)		<0.001	20 (17.9)		102 (1.0)		21.2 (12.6–35.7)		<0.001
ISS AOR	-		-		2.2 (1.1–4.5)		0.036	-		-		6.2 (3.2–11.9)		<0.001

**Table 7:** Management characteristics of hospitalized trauma patients who had stable SBP but an RSI < 1.

Variables	RSI < 1, SBP ≥ 90 *n* = 186	RSI ≥ 1, SBP ≥ 90 *n* = 9809	OR (95% CI)	*p*
Procedures at ED				
Intubation	17 (9.1)	177 (1.8)	5.5 (3.3–9.2)	<0.001
Chest tube insertion	16 (8.6)	125 (1.3)	7.3 (4.2–12.5)	<0.001
Blood transfusion	29 (15.6)	217 (2.2)	8.2 (5.4–12.4)	<0.001
Hospital LOS				
days	16.6 ± 19.0	9.0 ± 9.8	-	
ICU LOS				
n (%)	81 (43.5)	1496 (15.3)	4.3 (3.2–5.8)	<0.001
days	13.2 ± 14.9	8.7 ± 10.3	-	<0.001

## Appendix

**Table A1:** The detailed data regarding patient number, percentage, odds ratio, and *p*-value of the associated injuries of hospitalized trauma patients as illustrated in the figure.

Variables	(A)RSI ≥ 1 (L)RSI < 1 *n* = 76 (II)	(A)RSI < 1 (L)RSI ≥ 1 *n* = 251 (III)	(A)RSI < 1 (L)RSI < 1 *n* = 80 (IV)	(A)RSI ≥ 1 (L)RSI ≥ 1 *n* = 9827 (I)	II *vs.* I		III *vs.* I		IV *vs.* I	
					OR (95%CI)	*p*	OR (95%CI)	*p*	OR (95%CI)	*p*
**Head/Neck trauma**										
**Cranial fracture**	13 (17.1)	23 (9.2)	13 (16.2)	554 (5.6)	3.5 (1.9–6.3)	<0.001	1.7 (1.1−2.6)	0.018	3.2 (1.8−5.9)	**<0.001**
**Epidural hematoma**	11 (14.5)	12 (4.8)	9 (11.2)	352 (3.6)	4.6 (2.4−8.7)	<0.001	1.4 (0.8−2.4)	0.315	3.4 (1.7−6.9)	**<0.001**
**Subdural hematoma**	15 (19.7)	18 (7.2)	13 (16.2)	709 (7.2)	3.2 (1.8−5.6)	<0.001	1.0 (0.6−1.6)	0.979	2.5 (1.4−4.5)	**0.002**
**Subarachnoid hemorrhage**	17 (22.4)	33 (13.1)	6 (7.5)	803 (8.2)	3.2 (1.9−5.6)	<0.001	1.7 (1.2−2.5)	0.005	0.9 (0.4−2.1)	**0.827**
**Intracerebral hematoma**	2 (2.6)	7 (2.8)	5 (6.2)	159 (1.6)	1.6 (0.4−6.8)	0.486	1.7 (0.8−3.8)	0.150	4.1 (1.6−10.2)	**0.001**
**Cerebral contusion**	12 (15.8)	11 (4.4)	5 (6.2)	440 (4.5)	4.0 (2.1−7.5)	<0.001	1.0 (0.5−1.8)	0.943	1.4 (0.6−3.5)	**0.446**
**Cervical vertebral fracture**	3 (3.9)	8 (3.2)	5 (6.2)	86 (0.9)	4.7 (1.4−15.1)	0.005	3.7 (1.8−7.8)	<0.001	7.6 (3.0−19.1)	**<0.001**
**Face trauma**										
**Orbital fracture**	2 (2.6)	0 (0.0)	1 (1.2)	199 (2.0)	1.3 (0.3−5.4)	0.709	-	0.023	0.6 (0.1−4.4)	**0.624**
**Nasal fracture**	2 (2.6)	3 (1.2)	2 (2.5)	109 (1.1)	2.4 (0.6−9.9)	0.209	1.1 (0.3−3.4)	0.898	2.3 (0.6−9.4)	**0.239**
**Maxillary fracture**	11 (14.5)	21 (8.4)	6 (7.5)	621 (6.3)	2.5 (1.3−4.8)	0.004	1.4 (0.9−2.1)	0.190	1.2 (0.5−2.8)	**0.666**
**Mandibular fracture**	4 (5.3)	8 (3.2)	4 (5.0)	230 (2.3)	2.3 (0.8−6.4)	0.095	1.4 (0.7−2.8)	0.383	2.2 (0.8−6.1)	**0.119**
**Thoracic trauma**										
**Rib fracture**	15 (19.7)	41 (16.3)	15 (18.8)	811 (8.3)	2.7 (1.6−4.8)	<0.001	2.2 (1.5−3.1)	<0.001	2.6 (1.5−4.5)	**0.001**
**Hemothorax**	6 (7.9)	12 (4.8)	7 (8.8)	144 (1.5)	5.8 (2.5−13.5)	<0.001	3.4 (1.9−6.2)	<0.001	6.4 (2.9−14.3)	**<0.001**
**Pneumothorax**	7 (9.2)	13 (5.2)	6 (7.5)	148 (1.5)	6.6 (3.0−14.7)	<0.001	3.6 (2.0−6.4)	<0.001	5.3 (2.3−12.4)	**<0.001**
**Hemopneumothorax**	7 (9.2)	17 (6.8)	10 (12.5)	114 (1.2)	8.6 (3.9−19.2)	<0.001	6.2 (3.7−10.5)	<0.001	12.2 (6.1−24.2)	**<0.001**
**Lung contusion**	8 (10.5)	7 (2.8)	4 (5.0)	16 (0.2)	72.14 (29.9−174.2)	<0.001	17.6 (7.2−43.2)	<0.001	32.3 (10.5−98.8)	**<0.001**
**Abdominal trauma**										
**Intra-abdominal injury**	1 (1.3)	18 (7.2)	6 (7.5)	156 (1.6)	0.8 (0.1−6.0)	0.850	4.8 (2.9−7.9)	<0.001	5.0 (2.2−11.7)	**<0.001**
**Hepatic injury**	12 (15.8)	23 (9.2)	13 (16.2)	155 (1.6)	11.7 (6.2−22.1)	<0.001	6.3 (4.0−9.9)	<0.001	12.1 (6.6−22.4)	**<0.001**
**Splenic injury**	3 (3.9)	15 (6.0)	8 (10.0)	75 (0.8)	5.3 (1.6−17.3)	0.002	8.3 (4.7−14.6)	<0.001	14.4 (6.7−31.1)	**<0.001**
**Renal injury**	1 (1.3)	4 (1.6)	5 (6.2)	42 (0.4)	3.1 (0.4−22.9)	0.241	3.8 (1.3−10.6)	0.007	15.5 (6.0−40.4)	**<0.001**
**Extremity trauma**										
**Humeral fracture**	10 (13.2)	7 (2.8)	6 (7.5)	154 (1.6)	9.5 (4.8−18.9)	<0.001	1.8 (0.8−3.9)	0.127	5.1 (2.2−11.9)	**<0.001**
**Radial fracture**	12 (15.8)	14 (5.6)	8 (10.0)	1015 (10.3)	1.6 (0.9−3.0)	0.120	0.5 (0.3−0.9)	0.014	1.0 (0.5−2.0)	**0.923**
**Ulnar fracture**	4 (5.3)	19 (7.6)	7 (8.8)	488 (5.0)	1.1 (0.4−2.9)	0.905	1.6 (1.0−2.5)	0.062	1.8 (0.8−4.0)	**0.122**
**Pelvic fracture**	8 (10.5)	28 (11.2)	17 (21.2)	244 (2.5)	4.6 (2.2−9.7)	<0.001	4.9 (3.3−7.5)	<0.001	10.6 (6.1−18.4)	**<0.001**
**Femoral fracture**	13 (17.1)	34 (13.5)	16 (20.0)	769 (7.8)	2.4 (1.3−4.4)	0.003	1.8 (1.3−2.7)	0.001	2.9 (1.7−5.1)	**<0.001**
**Tibia fracture**	8 (10.5)	38 (15.1)	6 (7.5)	712 (7.2)	1.5 (0.7−3.2)	0.272	2.3 (1.6−3.3)	<0.001	1.0 (0.5−2.4)	**0.930**
**Fibular fracture**	6 (7.9)	22 (8.8)	5 (6.2)	392 (4.0)	2.1 (0.9−4.8)	0.084	2.3 (1.5−3.6)	<0.001	1.6 (0.7−4.0)	**0.304**

## Abbreviations

The following abbreviations are used in this manuscript:

AIS	Abbreviated injury scale
CI	Confidence interval
DCR	Damage control resuscitation
ED	Emergency department
GCS	Glasgow coma scale
HR	Heart rate
ICU	Intensive care unit
ISS	Injury severity score
LOS	Length of stay
OR	Odds ratio
RSI	Reverse shock index
SI	Shock index
SBP	Systolic blood pressure
